# Supplementary material for: Argininosuccinic aciduria fosters neuronal nitrosative stress reversed by Asl gene transfer
Source: Nat Commun. 2018 Aug 29;9:3505. doi: 10.1038/s41467-018-05972-1 (PMC6115417; doi:10.1038/s41467-018-05972-1)
Supplement: Supplementary file 1 — Supplementary information [file 41467_2018_5972_MOESM1_ESM.docx]

**SUPPLEMENTARY INFORMATION**

**-**

**ARGININOSUCCINIC ACIDURIA FOSTERS NEURONAL NITROSATIVE STRESS REVERSED BY *Asl* GENE TRANSFER**

**BARUTEAU *et al*.**

**
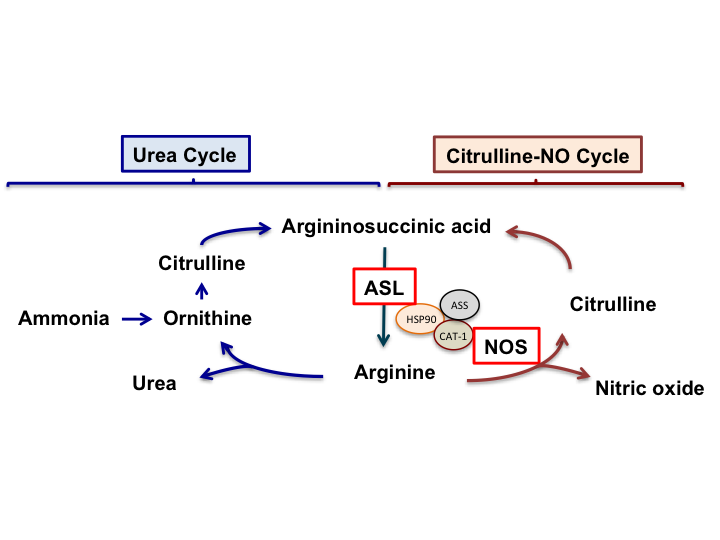
**

**Supplementary Figure 1. Metabolic pathways involving argininosuccinate lyase.** Argininosuccinate lyase (ASL) belongs to the urea cycle, which detoxifies ammonia into urea, and the citrulline-nitric oxide cycle, which allows nitric oxide (NO) production from nitric oxide synthase (NOS). All enzymes required in the urea cycle are expressed in the liver. The citrulline-NO cycle is expressed in most organs. ASL elicits (i) an enzymatic function in catalysing the production of arginine and fumarate from argininosuccinic acid, and (ii) a structural role required to maintain a multiprotein complex, which includes NOS, argininosuccinate synthase (ASS), a cationic amino acid transporter (CAT-1) and heat shock protein 90 (HSP90).

**
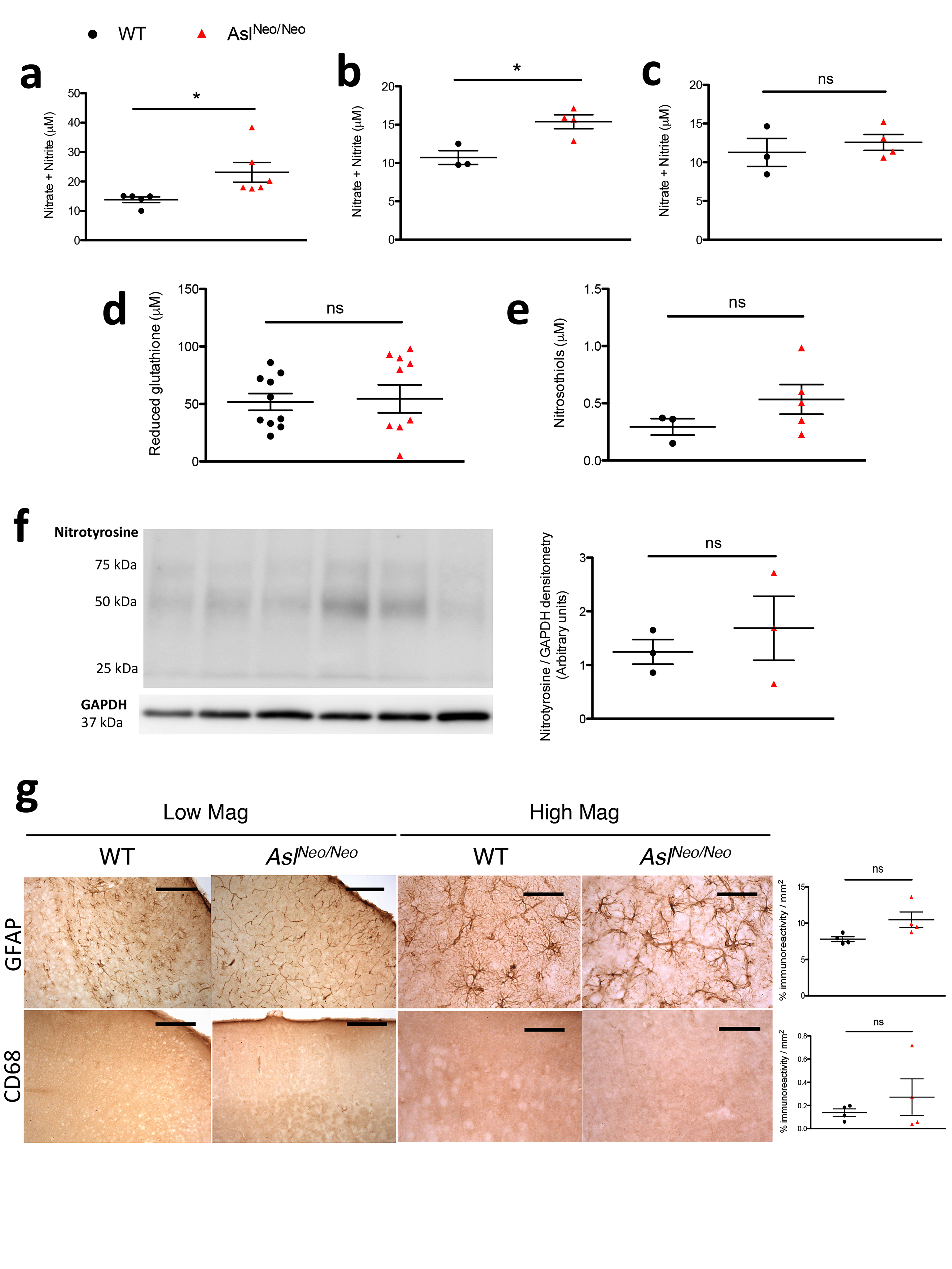
**

**Supplementary Figure 2. Nitrosative stress is predominant in forebrain and midbrain and is not caused by neuroinflammation.** Nitrite/nitrate levels were significantly increased in **(a)** the cerebrum, **(b)** the diencephalon and the midbrain but not **(c)** the hindbrain of *Asl^Neo/Neo^* mice compared to WT. (**d)** Reduced glutathione (n=9-10) levels, **(e)** nitrosothiols levels and **(f)** western blot analysis of nitrotyrosine-modified proteins in brain homogenates showed no difference between WT and *Asl^Neo/Neo^* mice (n=3). (g) Immunostaining for the astrocytic GFAP or microglial CD68 markers showed no difference between WT and *Asl^Neo/Neo^* mice (n=3). Experiments in 1-3 month-old mice. Horizontal lines display the mean ± SEM. Unpaired 2-tailed Student’s *t* test * p<0.05, ** p<0.01, *** p<0.001.

**
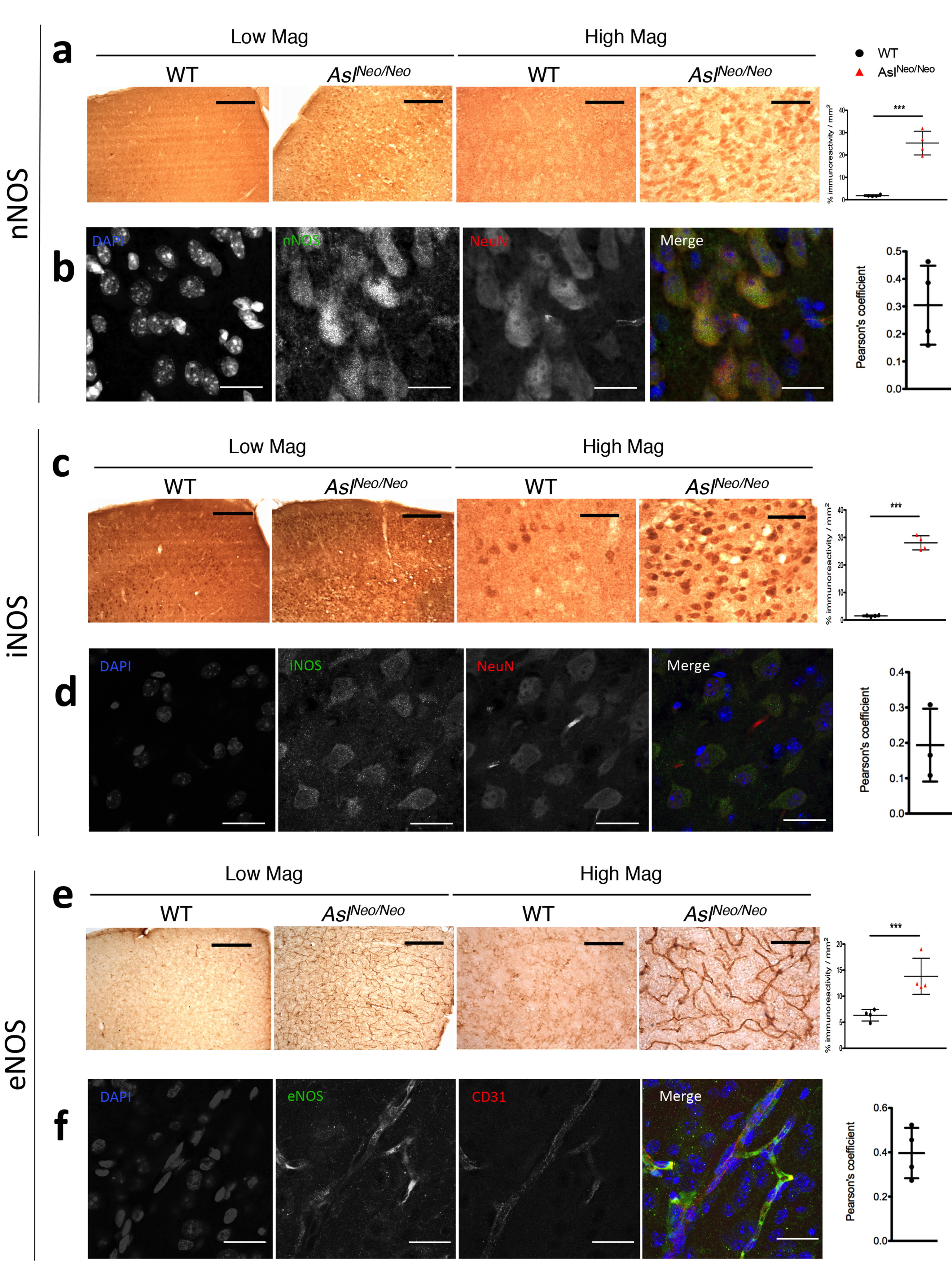
**

**Supplementary Figure 3. Increase immunostaining of NOS isoforms in the cortex of *Asl^Neo/Neo^* mice compared to WT. (a)** Significant increase of nNOS by immunohistochemistry **(b)** and by immunofluorescence in neurons. Merge panel show nNOS (green) and the neuronal marker NeuN (red) with nuclei counterstained with DAPI (blue). **(c)** Significant increase of iNOS by immunohistochemistry **(d)** and by immunofluorescence in neurons. **(e)** Significant increase of eNOS by immunohistochemistry **(f)** and by immunofluorescence in endothelial cells. **(a,c,e)** Analysis of cortical slices of *Asl^Neo/Neo^* and WT mice aged 1-3 month-old, n=3. Horizontal lines display the mean ± SEM. Unpaired 2-tailed Student’s *t* test *** p<0.001. **(b,d,f)** Colocalization between nNOS, iNOS, eNOS and NeuN was measured by the Pearson’s coefficient. Scale bars: **(a,c,e)** low and high magnification: 500 and 125 μm, respectively; **(b,d,f)** 25 μm. Figures show representative images. Low Mag: Low magnification. High Mag: High magnification.

**Supplementary Figure 4. Morphological comparison of brains showed no difference between WT and *Asl^Neo/Neo^* mice.** n=3, aged 4 months, scale bar 5 mm.

**Supplementary Figure 5. Biodistribution after AAV8.EFS.*GFP* neonatal intravenous injection. (a)** GFP fluorescence and **(b)** immunostaining in C57BL/6 pups receiving neonatal injection of AAV8.EFS.*GFP* (GFP) versus uninjected controls littermates. **(c)** GFP ELISA in peripheral organs in GFP injected pups. **(d)** qPCR in liver of GFP injected pups versus uninjected controls. Horizontal lines display the mean ± SEM. Unpaired 2-tailed Student’s *t* test *** p<0.001. Scale bars: **(a)** 5 mm; **(b)** 500 μm and 125 μm in main and inset pictures, respectively; n=4. GFP: Green Fluorescent Protein. Figures show representative images.

**
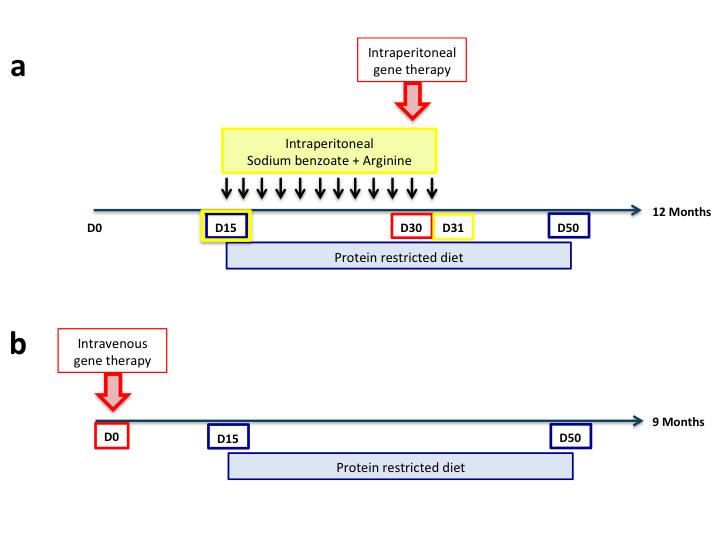
**

**Supplementary Figure 6. Experimental design for gene therapy intervention. (a)** Adult-injected *Asl^Neo/Neo^* mice received a supportive treatment consisting of a protein-restricted diet from day 15 to day 50, combined with daily intraperitoneal administration of an ammonia scavenger drug (sodium benzoate, 100 mg/kg/d) and arginine supplementation (1 g/kg/d) which was initiated at day 10 and halted at day 31, 24 hours after gene therapy injection. Untreated *Asl^Neo/Neo^* mice were treated identically except without gene therapy injection at day 30. Mice were monitored for 12 months. **(b)** Neonatally-injected *Asl^Neo/Neo^* mice received the same dietetic regimen without sodium benzoate and arginine administration. Mice were monitored for 9 months. WT mice received the same dietary regimen and water supplementation.

**
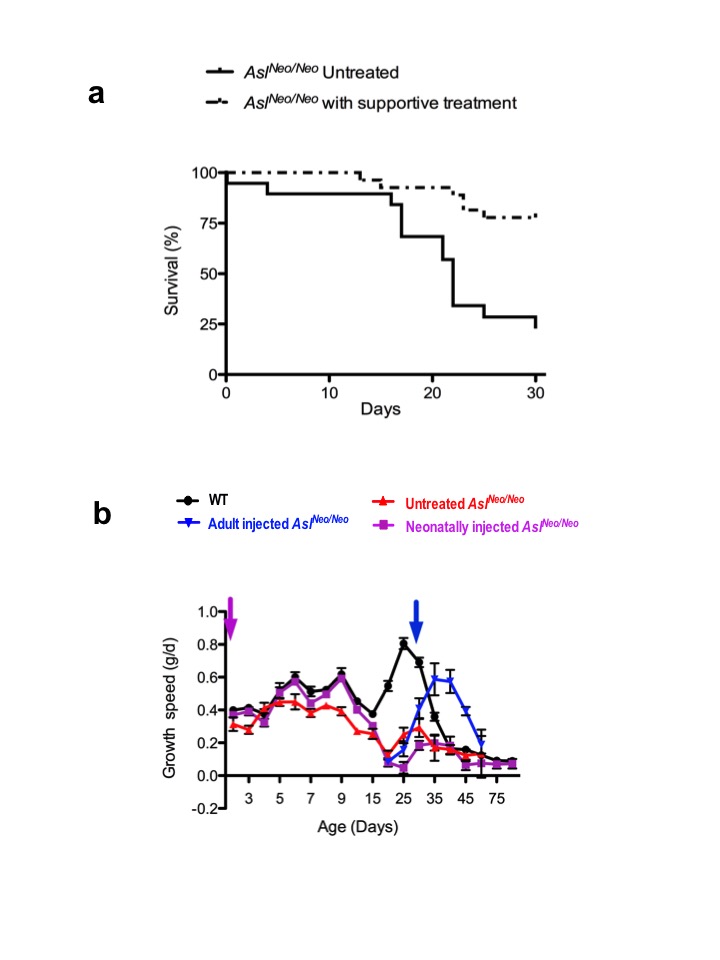
**

**Supplementary Figure 7. Systemic injection of AAV8.EFS.*mAsl* *mAsl* shows a sustained improvement in the macroscopic phenotype of adult-injected *Asl^Neo/Neo^*** **mice but only a transient improvement after neonatal injection. (a)** Survival curve of *Asl^Neo/Neo^* mice untreated or receiving supportive treatment during the first month of life. Log-rank test *p*=0.0025; n=18-19. **(b)** Modification of the growth speed after systemic injection of gene therapy at day 0 or 30 (purple and blue arrow respectively) compared to WT and untreated *Asl^Neo/Neo^* mice. WT n=19; untreated *Asl^Neo/Neo^* n=19; adult-injected *Asl^Neo/Neo^* n=5; neonatally-injected *Asl^Neo/Neo^* n=5.

**Supplementary Figure 8. Correction of the fur phenotype in adult *Asl^Neo/Neo^* mice after gene therapy. (a)** 30-days old *Asl^Neo/Neo^* mouse before (left panel) and 15 days after (right panel) a single intraperitoneal injection of AAV8.EFS.*mASL* vector. **(b-d)** Representative images of hair of a 3 month-old WT, untreated and adult-injected *Asl^Neo/Neo^* mouse. Scale bars: **(b, d)** 125 μm; **(c)** 500 μm; n=3.

**Supplementary Figure 9. Correction of the urea cycle after gene therapy. (a)** Concentration of plasma ammonia, **(b)** argininosuccinic acid, **(c)** L-citrulline and **(d)** L-arginine from dried blood spots in 2 month-old mice. **(e)** Orotic aciduria in 3 and 10 week-old mice. **(f)** Plasma alanine amino transferase (ALT) in 9-12 month-old mice. **(g)** Vector genome copy number in liver samples from untreated and treated *Asl^Neo/Neo^* mice at harvest (n=4-5). One-way ANOVA with Dunnett’s post test compared to WT **(a, b, c, d, f)** and untreated *Asl^Neo/Neo^* mice **(e)**; Bonferroni post test **(g)**; ** p<0.01, *** p<0.001, ns not significant. **(a, b, c, d, f)**: WT n=15-17; untreated *Asl^Neo/Neo^* n=9-18; adult-injected *Asl^Neo/Neo^* n=5; neonatally-injected *Asl^Neo/Neo^* n=5.

**
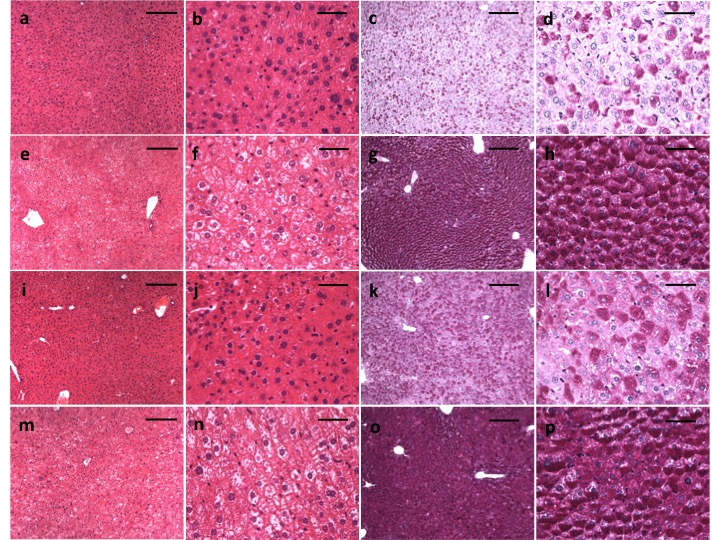
**

**Supplementary Figure 10. Normalisation of the microscopic liver architecture in adult *Asl^Neo/Neo^* mice after gene therapy.** Representative images of liver from **(a-d)** WT, **(e-h)** untreated, **(i-l)** adult-injected and **(m-p)** neonatally-injected *Asl^Neo/Neo^* mice stained with haematoxylin and eosin (H&E) highlighting vacuolar cytoplasmic deposits and periodic acid Schiff (PAS) demonstrating glycogen deposits. Scale bars are **(a, c, e, g, I, k, m, o)** 500 μm and **(b, d, f, h, j, l, n, p)** 125 μm respectively; n=3 per group. Only the 3 animals with the highest ASL activity were studied for adult- and neonatally-injected groups.

**Supplementary Figure 11. The hepatic impairment of NO metabolism showed a sustained improvement in adult *Asl^Neo/Neo^* mice after gene therapy. (a)** Nitrite/nitrate levels and **(b)** reduced glutathione (GSH) in liver of WT (n=8-10), untreated (n=8-10), adult-injected (n=4-5) and neonatally-injected *Asl^Neo/Neo^* mice (n=4-5). Horizontal lines display the mean ± SEM. One-way ANOVA with Dunnett’s post test compared to WT: ns - not significant, * p<0.05, ** p<0.01, *** p<0.001.


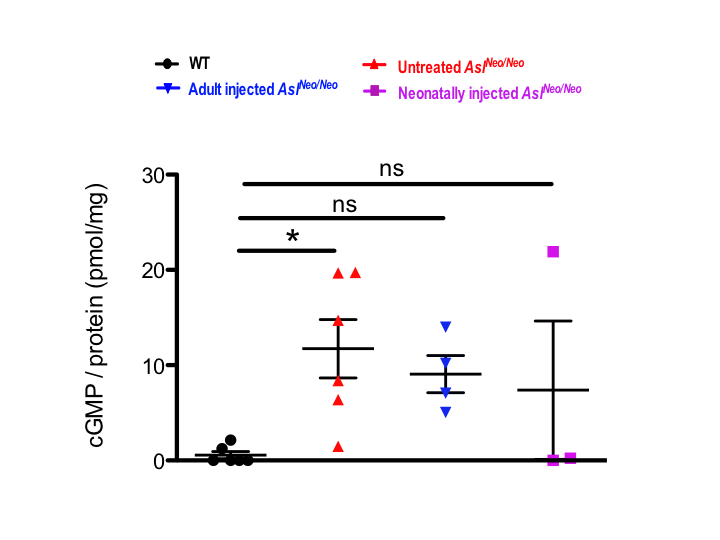


**Supplementary Figure 12. Increased cGMP levels in brain homogenates were not corrected after gene therapy.** cGMP levels of brain homogenates of WT (n=6), untreated (n=6), adult-injected (n=4) and neonatally-injected *Asl^Neo/Neo^* mice (n=3). Horizontal lines display the mean ± SEM. One-way ANOVA with Dunnett’s post test compared to untreated *Asl^Neo/Neo^* mice: ns - not significant, * p<0.05.

**Supplementary Figure 13. Uncropped western blot corresponding to Supplementary Figure 2. (a)** Nitrotyrosine and **(b)** GAPDH immunoblots.
